# Complete chloroplast genome sequence of the medical fern *Drynaria roosii* and its phylogenetic analysis

**DOI:** 10.1080/23802359.2016.1275835

**Published:** 2017-01-09

**Authors:** Meiyu Sun, Jingrui Li, Dong Li, Lei Shi

**Affiliations:** Key Laboratory of Plant Resources and Beijing Botanical Garden, Institute of Botany, Chinese Academy of Sciences, Beijing, China

**Keywords:** Chloroplast genome, *Drynaria roosii*, *de novo* sequencing, phylogenetic relationship

## Abstract

In this study, the complete chloroplast genome of the medical fern *Drynaria roosii* was completed and analyzed in order to understand the evolution of the genome of the fern lineages. In *D. roosii*, the circular double-stranded cpDNA sequence of 154,305 bp consists of two inverted repeat (IRA and IRB) regions of 23,416 bp each, a large single-copy (LSC) region of 86,040 bp and a small single-copy (SSC) region of 21,433 bp. The overall GC content is 40.92% and the GC contents of LSC, IRs, and SSC are 39.75%, 45.07%, and 36.60%, respectively. *D. roosii* with 108 annotated unique genes included 85 protein-coding genes, 19 tRNA genes, and 4 rRNA genes. Using the whole chloroplast genome sequences alignment of 18 species from ferns, the phylogenetic relationship was built. The phylogenetic position of *D. roosii* was closely clustered with *Adiantum capillus-veneris*, *Cheilanthes lindheimeri,* and *Pteridium aquilium* subsp*. Aquilinum* as sister species and then clustered with *Alsophila spinulosa*, *Lygodium japonicum*, *Diplopterygium glaucum*, and *Osmundastrum cinnamomeum*. *D. roosii* belongs to *Polypodiales*. The complete chloroplast genome of *D. roosii* provides utility information for ferns evolutionary and genomic studies.

## Introduction

*Drynaria roosii* widely distributed in south-east and south-west of China, also called *D. fortunei* (Kunze) J. Sm, is applied extensively as Drynaria Rhizome (Gusuibu) in traditional Chinese medicine (TCM) (Zhang et al. 2009; Yang et al. 2010; Li et al. 2011). ‘Qianggu capsule’, which is made using Gusuibu as the main material, fills the gap in treating osteoporosis with Category II New Drug of TCM in China (Xie et al. 2004). Moreover, the advantage of ferns’ chloroplast genomes in moderate size (∼131–168 kb) makes it to be easily sequenced, resulting in its wide use in genomics and phylogenetic studies (Wolf et al. 2003; Roper et al. 2007; Gao et al. 2009; Kim et al. 2014; Zhong et al. 2014; Lu et al. 2015). In this study, we characterized the complete chloroplast genome sequence of *D. roosii* to contribute to further physiological, molecular, and phylogenetical studies of this plant.

Sample of *D. roosii* was collected from Emei mount, Sichuan Province of China, and maintained in Beijing Botanical Garden (NSII accession number 12245, http://www.nsii.org.cn/node/79/cvh/33/14b/4838354). Genomic DNA was extracted following the modified CTAB DNA extraction protocol (Attitalla et al. 2011) and then subjected to build up genomic library and pair-end sequencing (2 × 150 bp) by MiSeq (Illumina, San Diego, CA). Approximately 1,142 Mb of raw data and 1,039 Mb of clean data were obtained, and *de novo* assembled by the SOAPdenovo software (Zhao et al. 2011) with about an average 278 × coverage. Compared with the chloroplast sequence of *Adiantum capillus-veneris* (NC_004766) as a reference, 168 representative chloroplast contigs were selected and joined into a single draft sequence. DOGMA software was used for annotation of protein-coding genes (PCGs) in the chloroplast genome (Wyman et al. 2004), and manually inspected to predict PCGs, transfer RNA (tRNA) genes, and ribosomal RNA (rRNA) genes.

The complete chloroplast genome of *D. roosii* had total sequence length of 154,305 bp and a higher GC content of 40.92% (GenBank accession number KY075853), which was featured with the conserved quadripartite structure of chloroplast containing a large single-copy region (LSC) with a size of 86,040 bp, a small single-copy region (SSC) with a size of 21,433 bp, and two inverted repeats (IRs, including IRA and IRB) each with the size of 23,416 bp, respectively. A total of 108 genes were annotated in *D. roosii* chloroplast genome composed by 85 PCRs, 19 tRNA genes, and 4 rRNA genes. Sixteen PCRs contained introns with two of these genes (clpP and ycf3) exhibiting two introns and the rest of the genes contain a single intron. Seven PCRs had two copies and two PCRs had three copies. In the two IR regions, 16 genes were duplicated including 7 PCRs, 5 tRNAs, and 4 rRNAs.

**Figure 1.**
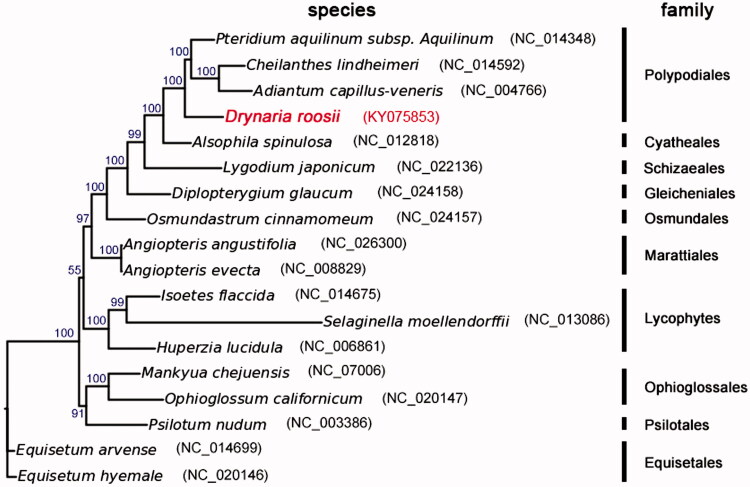
Molecular phylogeny of *Drynaria roosii* and other related species in ferns based on complete chloroplast genome. The complete chloroplast genome is downloaded from NCBI database and the phylogenic tree is constructed by MEGA6 software. The gene’s accession number for tree construction is listed as follows: *Pteridium aquilinum subsp. Aquilinum* (NC_014348), *Cheilanthes lindheimeri* (NC_014592), *Adiantum capillus-veneris* (NC_004766), *Alsophila spinulosa* (NC_012818), *Lygodium japonicum* (NC_022136), *Diplopterygium glaucum* (NC_024158), *Osmundastrum cinnamomeum* (NC_024157), *Angiopteris angustifolia* (NC_026300), *Angiopteris evecta* (NC_008829), *Isoetes flaccida* (NC_014675), *Selaginella moellendorffii* (NC_013086), *Huperzia lucidula* (NC_006861), *Mankyua chejuensis* (NC_017006), *Ophioglossum californicum* (NC_020147), *Psilotum nudum* (NC_003386), *Equisetum arvense* (NC_014699), *Equisetum hyemale* (NC_020146).

To validate the phylogenetic position of *D. roosii*, we used MEGA6 (Tamura et al. 2013) to construct a maximum-likelihood tree (with 500 bootstrap replicates) containing complete cpDNA of the other 17 species in ferns (Figure 1). The phylogenetic position of *D. roosii* was closely clustered with *A. capillus-veneris*, *Cheilanthes lindheimeri*, and *Pteridium aquilium* subsp. *Aquilinum* as sister species and then clustered with *Alsophila spinulosa*, *Lygodium japonicum*, *Diplopterygium glaucum*, and *Osmundastrum cinnamomeum*. *D. roosii* belongs to *Polypodiales*. In conclusion, the complete cpDNA of *D. roosii* is decoded for the first time in this study and provides essential and important DNA molecular data for further phylogenetic and evolutionary analysis for ferns.
